# A Unique Presentation of an Ectopic Pregnancy After Appendectomy

**DOI:** 10.7759/cureus.11560

**Published:** 2020-11-19

**Authors:** Benjamin E Grounds, Jennifer Cross

**Affiliations:** 1 Obstetrics and Gynecology, Naval Medical Center Portsmouth, Portsmouth, USA

**Keywords:** ectopic pregnancy, appendectomy, surgical staple

## Abstract

Ectopic pregnancies account for the majority of deaths in early pregnancy. A 27-year-old woman with unexplained infertility and a history of an appendectomy was referred to the reproductive endocrinology clinic. She was initially diagnosed with a pregnancy of unknown location and was later found to have an ectopic pregnancy. Final pathology found a surgical staple likely present from a previous appendectomy within the fallopian tube proximal to the ectopic site. The surgical staple is postulated to have entered the tube through ciliary action and caused the ectopic pregnancy. This rare case highlights physicians’ need to carefully monitor and remove visible loose staples after using automated stapling devices.

## Introduction

Ectopic pregnancies account for approximately 1% of all first-trimester pregnancies, yet they are responsible for most early pregnancy-related deaths and 3% of all pregnancy-related deaths. Inflammation within the fallopian tube is thought to cause damage that subsequently affects an embryo's transit through the tube. This leads to an embryo implanting in the fallopian tube and invading its muscularis layer [1].

The most common risk factors associated with ectopic pregnancy are a history of pelvic inflammatory disease, history of tubal surgery, prior ectopic pregnancy, smoking, history of medical or surgical abortion, three or more prior miscarriages, more than five-lifetime sexual partners, infertility, age older than 40 years, and intrauterine device use [1].

This article describes an ectopic pregnancy in a patient who had a prior appendectomy. A surgical staple was identified within the fallopian tube proximal to the ectopic. Upon review, no similar case has been previously reported in the literature.

## Case presentation

A 27-year-old woman with unexplained infertility was referred to the reproductive endocrinology clinic. Her medical history was significant for polycystic ovary syndrome. She reported menstrual cycles every 30 to 37 days with menses lasting five to seven days. She was a nonsmoker and denied a history of sexually transmitted infections. Her past surgical history was significant for a laparoscopic appendectomy and right salpingectomy for ruptured appendicitis at age 13.

As part of the infertility workup, a saline infusion sonohysterogram was ordered but not completed due to a positive pregnancy test and pelvic cramping upon presentation to radiology. The following day, she reported that her symptoms became worse to include passing large clots. She then presented to the emergency department, and her quantitative human chorionic gonadotropin (HCG) was 98 mIU/ml. A transvaginal ultrasound was performed, which showed no intrauterine pregnancy and normal adnexa. She was diagnosed with a pregnancy of unknown location and referred to the acute gynecology clinic.

Three days later, she presented to the clinic and reported vaginal spotting and that her pelvic pain had resolved. Her quantitative HCG was 449 mIU/ml. She was given return precautions and a follow-up appointment. Seven days later, she presented to the clinic for her scheduled follow-up assessment. Her quantitative HCG was now 3798 mIU/ml, and a bedside transvaginal ultrasound showed an ectopic pregnancy within her left adnexa with fetal cardiac activity. She was counseled, admitted to the hospital, and underwent an uncomplicated left salpingectomy.

**Figure 1:**
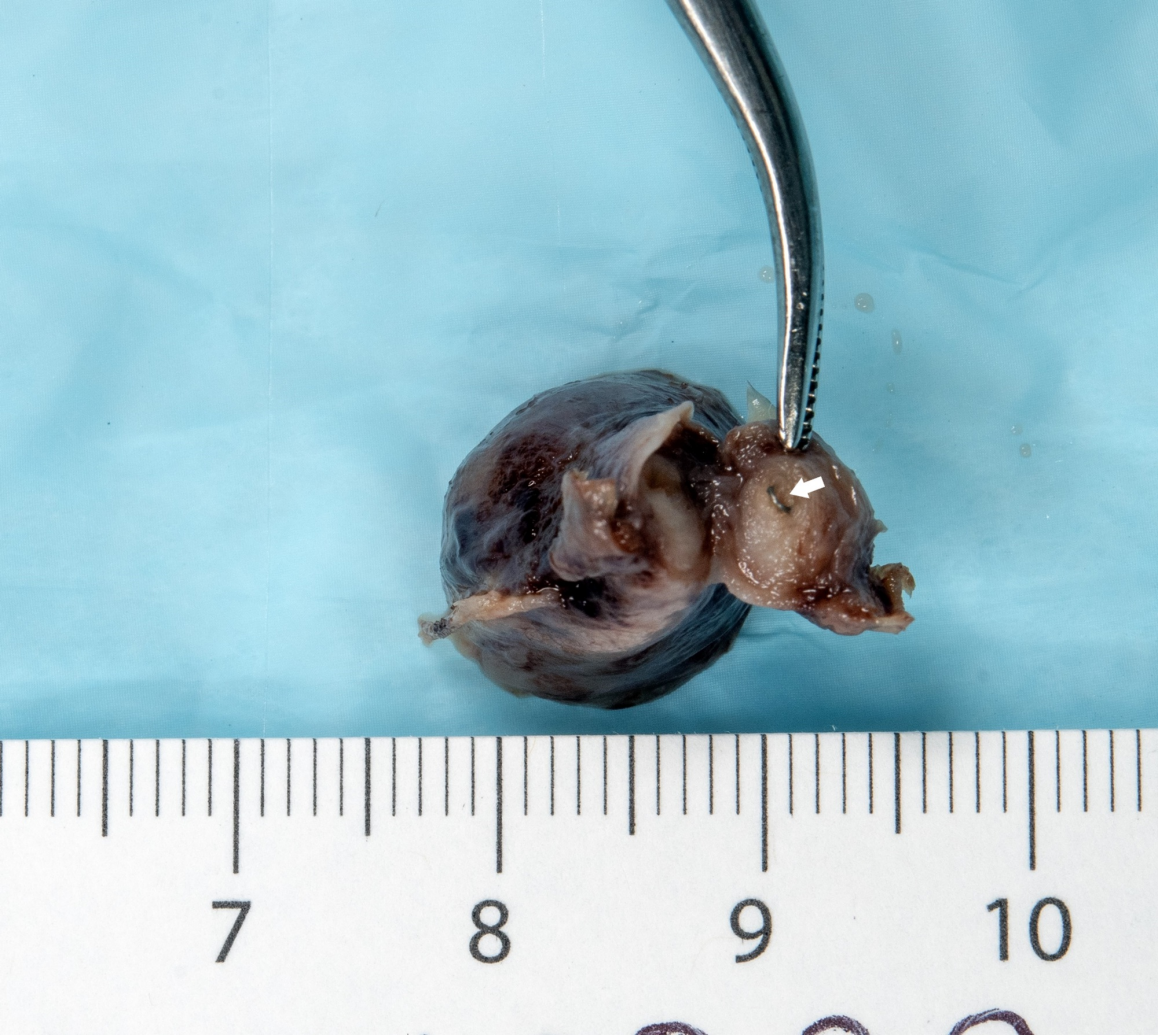
Picture of gross specimen with surgical staple noted in the fallopian tube.

**Figure 2:**
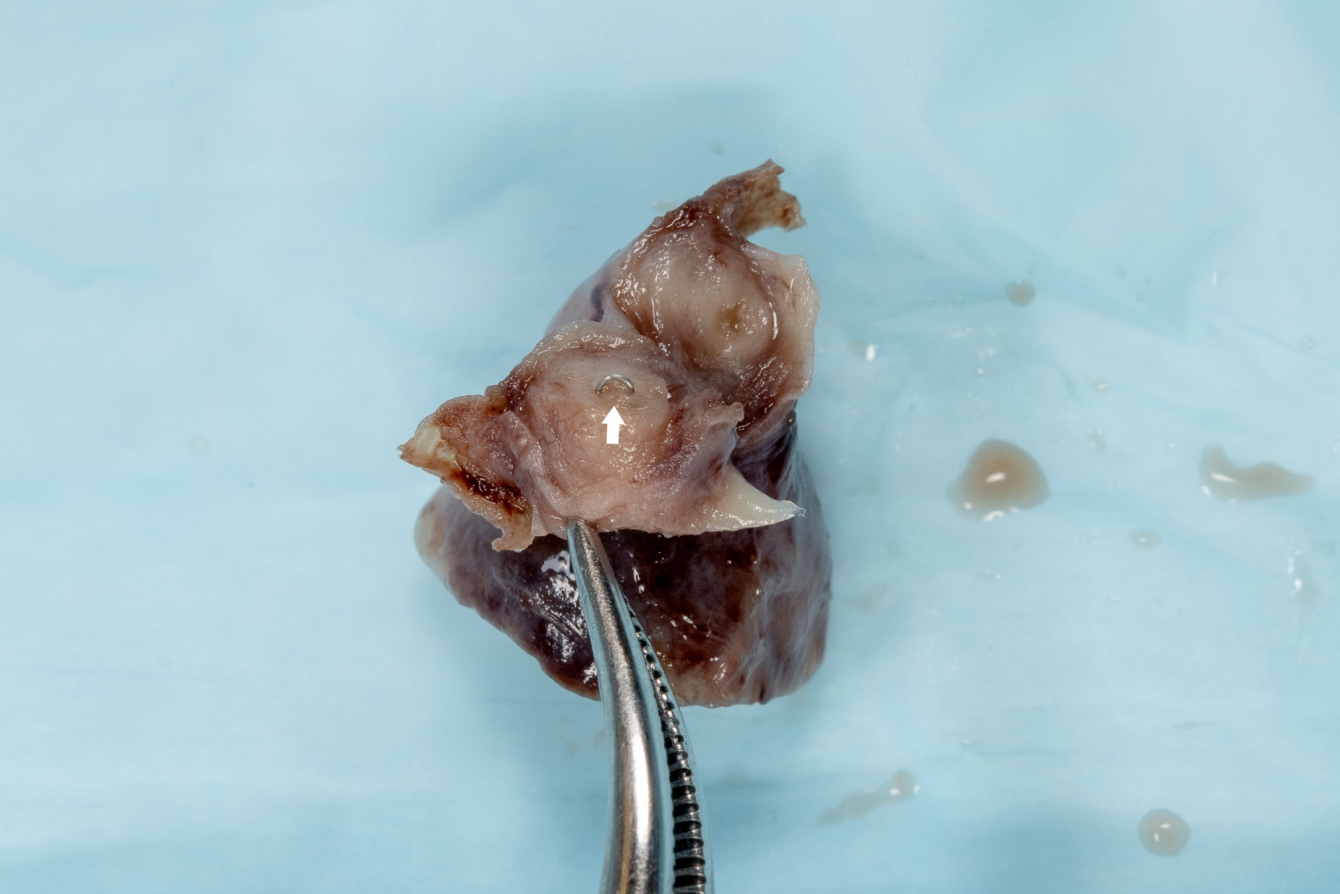
Additional image of surgical staple within the left fallopian tube.

Her final pathology evaluation results were consistent with ectopic pregnancy. Chorionic villi were identified within the fimbria and the wall of the fallopian tube. Proximal to the ectopic pregnancy, a single metal staple was found. The staple was embedded within the fallopian tube wall and believed to be from the prior appendectomy (Figures 1, 2). At the time of surgery, a few surgical staples were noted within the posterior cul-de-sac. 

## Discussion

The link between appendicitis and ectopic pregnancies has been evaluated; however, this is the first known report citing surgical staples as the possible cause of the ectopic pregnancy. Urbach and Cohen reviewed epidemiologic studies [2] and found an increased association between appendectomy and ectopic pregnancies with an odds ratio of 2.2, 95% CI: 1.6-3.2. The association in their study was not as strong when looking at appendiceal perforations, with an odds ratio of 1.7, 95% CI: 0.6-51. Their study was limited secondary to a paucity of data describing the relationship between ectopic pregnancies and prior appendicitis. Additionally, their study could not establish a causal relationship.

A meta-analysis by Elraiyah et al. also attempted to look at the effect appendicitis had on the risk of future ectopic pregnancies [3]. They found that a prior appendectomy significantly increased the risk of a future ectopic pregnancy with an odds ratio of 1.78, 95% CI: 1.46-2.16. This study was also limited by the fact that it was a retrospective study with limited data available.

Within the limited data available, it is hypothesized that inflammation from appendicitis might be a cause of the increased risk of future ectopic pregnancies. In this case, the proximal portion of the tube was also noted to have salpingitis isthmica nodosa. This inflammatory condition was noted in the tube near the staple.

No literature cites foreign bodies as a cause of this condition. It is also important to note that this patient differs from those documented in previous reports within the literature because a staple was found within the fallopian tube. We postulate that the staple was carried into the tube by the same ciliary action used to transport the oocyte after ovulation [4]. Automatic stapling devices are recognized as an acceptable method for closure of the appendix [5], and there are few references in the literature citing complications from loose staples [6,7].

## Conclusions

This case report describes a patient with an ectopic pregnancy likely caused by a stray surgical staple from a past appendectomy within the fallopian tube. History of appendicitis increases the risk of ectopic pregnancy; however, an ectopic pregnancy associated with a loose surgical staple has not previously been reported. This case report presents a significant motivation for physicians to carefully monitor and remove visible loose staples after using automated stapling devices.
